# PD98059 Influences Immune Factors and Enhances Opioid Analgesia in Model of Neuropathy

**DOI:** 10.1371/journal.pone.0138583

**Published:** 2015-10-01

**Authors:** Ewelina Rojewska, Katarzyna Popiolek-Barczyk, Natalia Kolosowska, Anna Piotrowska, Magdalena Zychowska, Wioletta Makuch, Barbara Przewlocka, Joanna Mika

**Affiliations:** Department of Pain Pharmacology, Institute of Pharmacology, Polish Academy of Sciences, Krakow, Poland; Toronto University, CANADA

## Abstract

Neuropathic pain treatment remains challenging due to ineffective therapy and resistance to opioid analgesia. Mitogen-activated protein kinase kinase (MAPKK) have been identified as the crucial regulators of pro- and antinociceptive factors. We used PD98059, an inhibitor of the MAPKK family members MEK1/2. The aim of study was to examine the influence of single and/or repeated PD98059 on nociception and opioid effectiveness in neuropathy. Moreover, we examined how PD98059 influences selected members of cellular pathways and cytokines. The PD98059 (2.5 mcg) was intrathecally preemptively administered before chronic constriction injury (CCI), and then once daily for 7 days. Additionally, at day 7 after CCI the PD98059-treated rats received a single injection of opioids. Using Western blot and qRT-PCR techniques in PD98059-treated rats we analyzed the mRNA and/or protein level of p38, ERK1/2, JNK, NF-kappaB, IL-1beta, IL-6, iNOS and IL-10 in the lumbar spinal cord. Our results indicate that PD98059 has an analgesic effects and potentiates morphine and/or buprenorphine analgesia. Parallel we observed that PD98059 inhibit upregulation of the CCI-elevated p38, ERK1/2, JNK and NF-kappaB protein levels. Moreover, PD98059 also prevented increase of pro- (IL-1beta, IL-6, and iNOS) but enhances anti-nociceptive (IL-10) factors. Summing up, PD98059 diminished pain and increased the effectiveness of opioids in neuropathy. The inhibition of MEKs might inactivate a variety of cell signaling pathways that are implicated in nociception.

## Introduction

Treatments for neuropathic pain are not satisfactory due to our incomplete understanding of its pathogenesis. Nuclear factor-kappaB (NF-kappaB) and Mitogen-Activated Protein Kinase (MAPK)-mediated pathways have been identified as the master regulators of pro- and antinociceptive factors. Studies have highlighted the role of the MAPKs in neuropathy [1, 2]. MAPKs are a family of serine/threonine protein kinases that transduce extracellular stimuli into intracellular transcriptional and posttranslational responses [3, 4]. This family consists of three major members that play a role in neuropathy: extracellular signal-regulated kinase (ERK), p38MAPK kinase (p38), and c-Jun N-terminal kinase (JNK) [5, 6]. In 1999, Ji et al. [7] reported the nociceptive-specific activation of ERK in spinal neurons; several years later, Zhuang et al. [8] showed that ERK is sequentially activated in neurons and glia after nerve injury. It seems that ERK is essential for the intracellular signaling that leads to the production of pronociceptive mediators [8]. Nerve injury causes phosphorylation of p38 [5, 9], which can increases the synthesis of proinflammatory mediators such as interleukin-1beta (IL-1beta) [9] and inducible nitric oxide synthase (iNOS) [10]. Intrathecal administration of p38 inhibitors (SB203580, FR167653, and minocycline) can diminish nerve injury-induced hyperalgesia and allodynia [11, 12, 13, 14, 15]. Our previously study demonstrated that minocycline diminished the level of proinflammatory factors, such as IL-6, IL-18, and MMP-9, during neuropathy [16] and enhanced morphine effectiveness [17, 18, 19]. In 2011 Lee et al. [6] showed that induction of p-NF-kappaB play important roles in trigeminal neuropathy and suggested that its blockade might be beneficial. Zhuang et al. [20] investigated the role of another MAPK family member, JNK, in neuropathic pain. After spinal nerve ligation (SNL) model, they observed persistent spinal activation of JNK in astrocytes. Intrathecal injection of D-JNKI-1JNK, a peptide inhibitor of JNK, reversed SNL-induced mechanical allodynia in rats. Additionally, Gao et al. [21] have demonstrated that activation of JNK is important for the chronic pain. A lot of studies have described a crucial role of NF-kappaB in pain models [22, 23, 24, 25, 26], and it is known that NF-kappaB is responsible for cytokine production [27]. In 2014 [25] we have already shown that administration of NF-kappaB inhibitor (parthenolide) as well as MEK1/2 inhibitor (U0126) diminished pain symptoms and enhanced morphine effectiveness in a rat model of neuropathy. Recently, it has been shown that during inflammation, intrathecal injection of PD98059 (MEK1/2 inhibitor) diminished the painful response to formalin injection in rats [7] and mice [28]. Zhuang et al. [8] showed that a single injection of PD98059 decreases spinal nerve ligation-induced mechanical allodynia.

The goal of our studies was to precisely determine how preemptive and then once daily for 7 days of PD98059 administration influences the development of neuropathic pain. Additionally, we examined using Western blot how PD98059 administration influences the CCI-elevated ERK, p38 and JNK protein levels. Moreover, we examined the influence of PD98059 on the spinal protein level of NF-kappaB and selected cytokines important for nociception transmission (IL-1beta, IL-6, IL-18, IL-10, iNOS). Additionally, we examined the possible influence of intrathecal single and repeated PD98059 administration on the effectiveness of morphine and/or buprenorphine in a rat model of neuropathy.

## Materials and Methods

### Animals

The rats (male Wistar, 300–350 g) were housed in cages with sawdust under a light/dark cycle (12/12 h, lights on at 08:00 h). Food and water were available without restrictions. All experiments were conducted according to the recommendations of IASP [29] and the NIH Guide for the Care and Use of Laboratory Animals. Experimental protocols were approved by the II Local Bioethics Committee from the National Ethics Committee for Experiments on Animals, at the Institute of Pharmacology, Polish Academy of Sciences (Cracow, Poland).

### Surgery for intrathecal implantation of catheters

Catheters for intrathecal (*i*.*t*.) injection were implanted according to Yaksh and Rudy [30] and our earlier publications [16, 19, 31]. The 7.8 cm long catheter (PE 10, outside diameter of 0.4 mm, Intramedic; Clay Adams, Parsippany, NJ) had been washed by 70% (v/v) ethanol and flushed with sterile water for injection before implantation. Head of rats under pentobarbital anaesthesia (60 mg/kg *i*.*p*.) was fixed on a stereotaxic table (David Kopf Instruments, Tujunga, CA), and the atlanto-occipital membrane was incised. The catheter was slowly introduced into the subarachnoid space until the end of the cannula reaches the rostral level of the spinal cord lumbar enlargement (L4–L5). The catheter was tightened after the injection of 10 mcl of water to flush the cannula. The first few days after catheter implantation the rats were monitored for physical impairments and rats exhibiting motor deficits were not included to the experiments. Experiment began a minimum of 1 week after the surgery. The injections through the *i*.*t*. catheter were made slowly (1–2 min) in a volume of 5 mcl, followed by a 10 mcl water for injection.

### Sciatic nerve injury

Chronic constriction injury (CCI) was made in rats under sodium pentobarbital anesthesia (60 mg/kg; intraperitoneal, *i*.*p*.) by four tied ligatures (4/0 silk) on the exposed right sciatic nerve. The ligatures with 1-mm spacing were made on nerve distal to the sciatic notch until a brief twitch in the respective limb. The procedure has been made according to Bennett and Xie [32] and was previously used in our studies [19, 16, 31]. After this type of nerve injury, all rats developed long-lasting allodynia and hyperalgesia which are characteristics of neuropathic pain.

### Behavioral study

#### von Frey test

An automatic von Frey apparatus (Dynamic Plantar Aesthesiometer Cat. No. 37400, Ugo Basile, Italy) was used to measure the degree of tactile allodynia. The animals were habituated in plastic cages with wire net floors 5 min before the experiment. The strengths of the von Frey stimuli ranged up to 26 g. The filament touch the midplantar surface of the ipsilateral paw and measurements were taken automatically as we described previously [16, 17, 25, 31]. No significant difference in paw reactions were noticed between the contralateral hind paw of CCI-exposed rats and control (naive) rats.

#### Cold plate test

The cold plate test (Cold/Hot Plate Analgesia Meter No. 05044, Columbus Instruments, USA) was used to measure the degree of thermal hyperalgesia as was previously described [16, 17, 25, 31]. The temperature of the cold plate was set at 5°C. The cut-off latency was 30 s. The time between putting the rat into the cold plate apparatus and the reaction (lifting the hind paw) was recorded. In the group of naive rats, the reaction of the first hind paw withdrawal was measured. In the group of CCI-exposed rats the injured paw was the first to react in all cases.

#### Tail-flick test

The pain threshold to a thermal stimuli in naïve rats was assessed by tail-flick latency evoked by a noxious hot stimuli, as determined by a tail-flick analgesic meter device (Tail Flick Analgesia Meter, IITC Life Science Inc., USA). The tail-flick test consisted of a beam of light focused on the dorsal tail surface 1 cm from the tip of the tail. The reaction time of the animal is thus determined and automatically recorded and the cut-off time for the tail-flick reaction was set to 9 s [18].

### Drug administration

PD98059 (2-(2-Amino-3-methoxyphenyl)-4H-1-benzopyran-4-one) (Sigma-Aldrich, USA) was dissolved in 75% DMSO. The PD98059 (2.5 mcg/5 mcl, *i*.*t*.) was single or repeated preemptively administered 16 h and 1 h before CCI and then once daily for 7 days (the administration was according to our previous paper [17], [25]). The Vehicle-treated CCI-exposed rats received 75% DMSO according to the same schedule. There was no significant difference in pain behavior between no-treated and V_(DMSO)_-treated CCI-exposed rats. This method of PD98059 or vehicle administration was used throughout the study and is referred to in the text as “repeated administration”. At day 7^th^ after CCI 30 min after PD98059 administration tactile allodynia was measured using von Frey test and thermal hyperalgesia was conducted using cold plate test. Additionally, at day 7^th^ after CCI the vehicle-treated and PD98059-treated rats received a single *i*.*t*. vehicle, morphine (2.5 mcg/5 mcl) or buprenorphine (2.5 mcg/5 mcl) injection 30 min after PD98059, and then 30 min later the von Frey and/or cold plate tests were repeated. Since the dose of morphine 2.5 mcg/5 mcl in naïve rats produced maximal analgesic effect in tail-flick test. We have used lower dose of morphine for co-administration experiments, so that we would be able to observe the possible enhancement of opioid effectiveness. The vehicle-treated and PD98059-treated naïve rats (uninjured rats) received a single *i*.*t*. vehicle, morphine (0.5 mcg/5 mcl) or buprenorphine (2.5 mcg/5 mcl) injection 30 min after PD98059, and then 30 min later the tail flick test was performed (S1 Table and S1 Fig).

### Quantitative reverse transcriptase polymerase chain reaction (qRT-PCR) analysis

Ipsilateral part of the dorsal part of the lumbar (L4–L6) spinal cord were collected immediately after decapitation (7 days after CCI and 4 hours after the last *i*.*t*. administration of PD98059). The tissue samples were placed in individual tubes containing the tissue storage reagent RNAlater (Qiagen Inc.) and were stored at -70°C for RNA isolation. Total RNA was extracted using the TRIzol reagent (Invitrogen). The RNA concentration was measured using a NanoDrop ND-1000 Spectrometer (NanoDrop Technologies). Reverse transcription was performed on 1 mcg of total RNA using Omniscript reverse transcriptase (Qiagen Inc.) at 37°C for 60 min. RT reactions were carried out in the presence of an RNAse inhibitor (rRNAsin, Promega) and an oligo (dT16) primer (Qiagen Inc.). cDNA was diluted 1:10 with H_2_O, and for each reaction, ~ 50 ng of cDNA synthesized from the total RNA of an individual animal was used for the quantitative real-time PCR (qRT-PCR) assay. qRT-PCR was performed using Assay-On-Demand TaqMan probes according to the manufacturer's protocol (Applied Biosystems), and the reactions were run on an iCycler device (BioRad, Hercules). The TaqMan primers used in experiments were as follow: Rn01527838_g1 (*Hprt*, hypoxanthine guanine rat hypoxanthine guanine phosphoribosyl transferase); Rn00580432_m1 (*IL-1beta*, interleukin 1 beta); Rn00561420_m1 (*IL-6*, interleukin 6); Rn01422083_m1 (*IL-18*, interleukin 18); Rn00561646_m1 (*iNOS*, inducible nitric oxide synthase); Rn00563409_m1 (*IL-10*, interleukin 10). The amplification efficiency for each assay (between 1.7 and 2) was determined by running a standard dilution curve. The cycle threshold values were calculated automatically by the iCycler IQ 3.0 software using the default parameters. RNA abundance was calculated as 2^-(threshold cycle)^. *HPRT* transcript levels do not significantly change in CCI-exposed rats, therefore, served as an adequate housekeeping gene (S2 Fig and S2 Table).

### Western blot analysis

Ipsilateral L4–L6 spinal cords were collected (7 days after CCI and 6 hours after the last *i*.*t*. administration of PD98059) in RIPA (Radio-Immunoprecipitation Assay) buffer with protease and phosphatase inhibitor cocktails (Sigma-Aldrich) and cleared by centrifugation (14000×g for 30 min, 4°C). The protein concentration in the supernatant was determined using the BCA Protein Assay Kit (Sigma-Aldrich). Samples containing 14 mcg of protein were heated in 4x Laemmli Sample Buffer 4x (250 mM Tris-HCl, pH 6.8, 4% LDS, 40% (w/v) glycerol, 0.02% bromophenol blue, and 15% beta-mercaptoethanol (Bio-Rad) for 5 min at 95°C and resolved by SDS—PAGE on 4–20% polyacrylamide gels (Criterion TGX, Bio-Rad). Following gel electrophoresis, the proteins were transferred by electroblotting to Immun-Blot PVDF membranes (Bio-Rad). Then, the transfer membranes were blocked for 1 h at 25°C using 5% non-fat dry milk (Bio-Rad) in Tris-buffered saline with 0.1% Tween 20 (TBST). Afterwards, the blots were incubated with the following anti-rat primary antibodies diluted in a Signal Boost Immunoreaction Enhancer Kit (Calbiochem) for 24 h at 4°C: p-p38 (Santa Cruz) 1:500; p-ERK1/2 (Santa Cruz) 1:500; p-JNK (Santa Cruz) 1:2000; p-NF-kappaB (Santa Cruz) 1:500; IL-1beta (Abcam) 1:1000; IL-6 (Invitrogen) 1:1000; iNOS (Sigma-Aldrich) 1:2000; IL-18 (R&D Systems) 1:1000; and IL-10 (Invitrogen) 1:2000. After four 5-minute washes in TBST, blots were incubated with anti-rabbit, anti-mouse or anti-goat secondary antibodies conjugated to horseradish peroxidase (HRP) diluted at 1:5000 in a Signal Boost Immunoreaction Enhancer Kit for 1 h at room temperature. After another four 5-minute washes in TBST, immunocomplexes were detected using Clarity Western ECL Substrate (Bio-Rad) and visualized with a Fujifilm Luminescent Image Analyzer LAS4000 System. The blots were washed 2 times for 5 minutes each in TBS, stripped using Restore Western Blot Stripping Buffer (Thermo Scientific), washed an additional 2 times for 5 minutes each in TBS, blocked and reprobed with an antibody against p38 (Santa Cruz) 1:500; ERK1/2 (Santa Cruz) 1:500; JNK (Santa Cruz) 1:2000; NF-kappaB (Santa Cruz) 1:500 and/or GAPDH 1:5000 (Millipore) as an internal loading control diluted in a Signal Boost Immunoreaction Enhancer Kit. During our procedures the membranes were used for multiple antibodies analysis and also were cut at the level, which allow us to investigate factors with different molecular weight (molecular weight are applied by the companies, which purchase the antibodies—more details is in Supporting information’s: S3 Table.), therefore we are not able to present full-length of immunoblots (S3 Fig). The relative levels of immunoreactivity were quantified by densitometry using Fujifilm Multi Gauge software. The immunoblots shown are representative of 4–8 individual samples.

### Data analysis

The behavioral data are presented as the mean ± S.E.M and the exact number of animals per group is placed on the description of the figures. The results of the experiments were evaluated using one-way analysis of variance (ANOVA). There was no significant difference between naïve and sham operated animals (e.g. 7 day in von Frey test values are as follows: naïve 25.5 g ± 0.3 and sham 25.1 g ± 0.5 and cold plate test values are as follows: naïve 28.0 s ± 0.8 and sham 28.5 s ± 0.9). The inter-group differences were analyzed with Bonferroni’s multiple comparison test. ***p<0.001 indicate significant differences compared to the control group (naïve rats); ^#^p<0.05, ^##^p<0.01 and ^###^p<0.001 indicate significant differences compared to the vehicle-treated CCI-exposed or naïve rats; ^$ $^p<0.01 and ^$ $ $^p<0.001 indicate significant differences compared to the repeated or single PD-treated CCI-exposed rats which received single morphine or buprenorphine injection; °p<0.05 and °°°p<0.001 indicate significant differences between repeated or single PD-treated CCI-exposed or naive rats to repeated or single PD-treated CCI-exposed or naive rats which received single morphine or buprenorphine injection.

The biochemical data are presented as the fold change of control (naïve rats) from mean ± S.E.M. of the ipsilateral sides of the dorsal lumbar spinal cord and the exact number of samples per group is placed on the description of the figures. There was no significant difference between naïve and sham operated animals e.g. for IL-18, IL-6, IL-1beta and iNOS protein level values are as follows: 1 ± 0.2 *vs* 0.9 ± 0.2; 1 ± 0.1 *vs*. 1.1 ± 0.1; 1 ± 0.1 *vs*. 1.12 ± 0.1; 1 ± 0.11 *vs*. 1.0 ± 0.1, respectively. The results for phosphorylated form of p38, ERK1/2, JNK and NF-kappaB were normalized to their total protein level in the same sample and were then expressed as a ratio of the average optical density values obtained for naïve animals. The inter-group differences were analyzed using one-way ANOVA followed by Bonferroni’s multiple comparison test. *p<0.05, **p<0.01 and ***p<0.001 indicate significant differences compared to the control group (naïve rats); ^#^p<0.05, ^##^p<0.01 and ^###^p<0.001 indicate significant differences compared to the vehicle-treated CCI-exposed rats.

All graphs were prepared using GraphPad Prism software (version 5.0).

## Results

### The effect of repeated *i*.*t*. administration of PD98059 on allodynia and hyperalgesia three and seven days after CCI in rats

**Fig 1:**
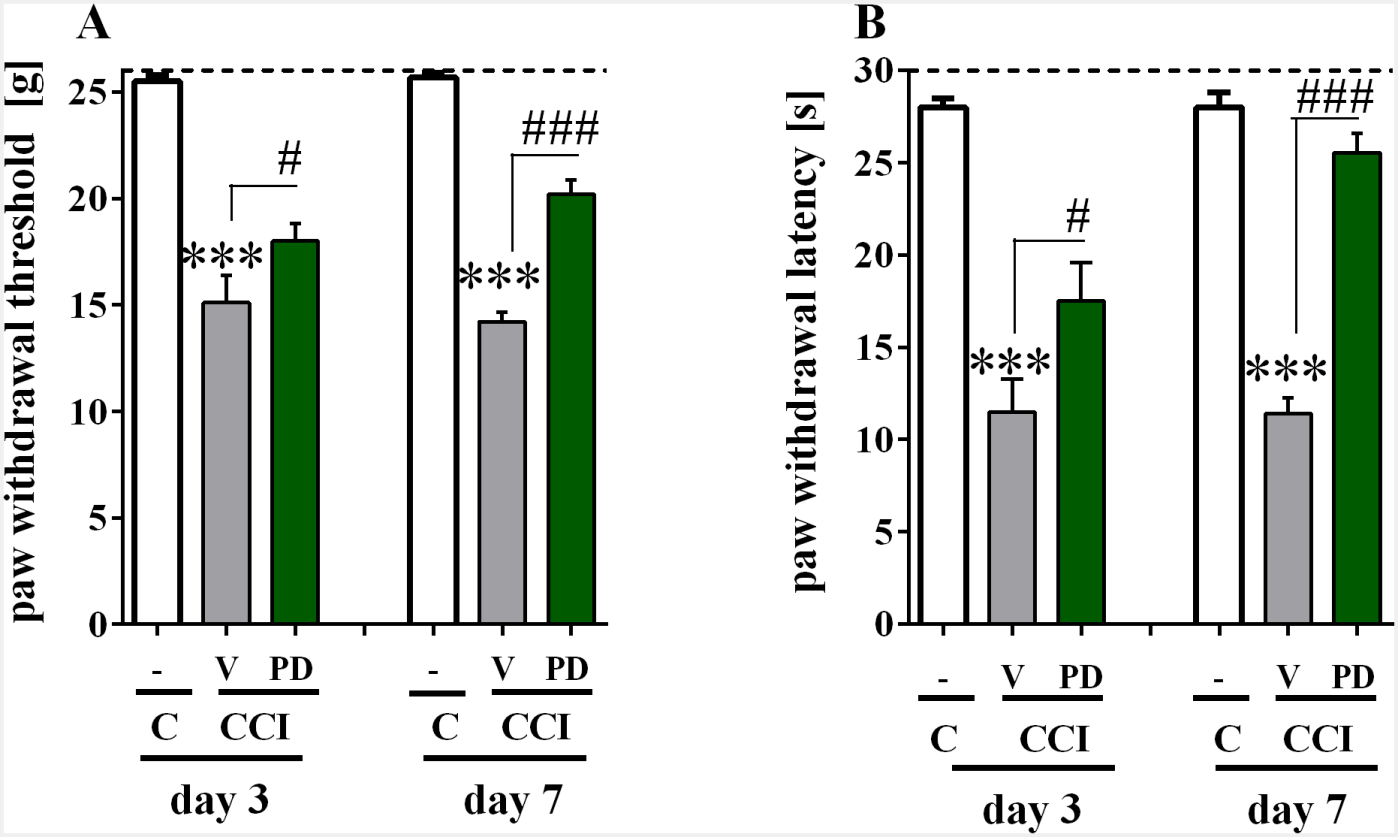
Effect of PD98059 on the development of neuropathic pain symptoms. Effect of PD98059 (2.5 mcg/5 mcl; *i*.*t*.; 16 h and 1 h before CCI and then once daily for 7 days) on the development of mechanical allodynia (A; von Frey test) and thermal hyperalgesia (B; cold plate) on days 3 and 7 after CCI. Pain was assessed using von Frey and cold plate tests 30 min after the last drug administration, and the data are presented as the means ± SEM. The inter-group differences were analyzed with ANOVA and Bonferroni’s multiple comparison test. ***p<0.001 indicates a significant difference compared to the control group (C), and ^#^p< 0.05 or ^###^p<0.001 indicates a significant difference compared to the V-CCI rats (ANOVA, Bonferroni’s test). The number of animals per group—day 3: C n = 7; V-CCI n = 7; PD-CCI n = 10; day 7: C n = 17; V-CCI n = 28; PD-CCI n = 26. C, control,; V, vehicle, PD, PD98059. The a line is drawn at value that represents the cut-off.

The influence of repeated *i*.*t*. administration of PD98059 (2.5 mcg), delivered at 16 h and 1 h before ligation of the sciatic nerve and then once daily for seven days, on mechanical allodynia and thermal hyperalgesia was measured three and seven days following CCI using von Frey (Fig 1A) and cold plate (Fig 1B) tests, respectively. In CCI-exposed rats the allodynia was measured three (15.1 g ± 1.3, n = 7) and seven (14.21 g ± 0.44, n = 28) days following CCI in comparison to the control rats (25.5 g ± 0.3, n = 7 and 25.7 g ± 0.2, respectively, n = 17) (Fig 1A). Simultaneously, the hyperalgesia was observed three (11.5 s ± 1.8, n = 7) and seven (11.4 s ± 0.88, n = 28) days following CCI in comparison to the control rats (28.0 s ± 0.5, n = 7 and 28.0 s ± 0.8, n = 17, respectively) (Fig 1B).

Repeated treatment with PD98059 attenuated mechanical allodynia measured by the von Frey test three (18.0 g ± 0.8, n = 10) and seven (20.21 g ± 0.67, n = 26) days after CCI in comparison to the vehicle-treated CCI-exposed rats (15.1 g ± 1.3, n = 7 and 14.21 g ± 0.44, n = 28, respectively) (Fig 1A). Furthermore, the repeated injections of PD98059 diminished thermal hyperalgesia, as was evaluated by the cold plate test, three (17.5 s ± 2.1, n = 10) and seven (25.54 s ± 1.03, n = 26) days following CCI compared to vehicle-treated CCI-exposed rats (11.5 s ± 1.8, n = 7 and 11.4 s ± 0.88, n = 28, respectively) (Fig 1B).

### The effect of repeated *i*.*t*. administration of PD98059 on the protein level of MAPK members (p38, ERK1/2, JNK) and NF-kappaB in the spinal cord on day seven after injury in CCI-exposed rats

**Fig 2:**
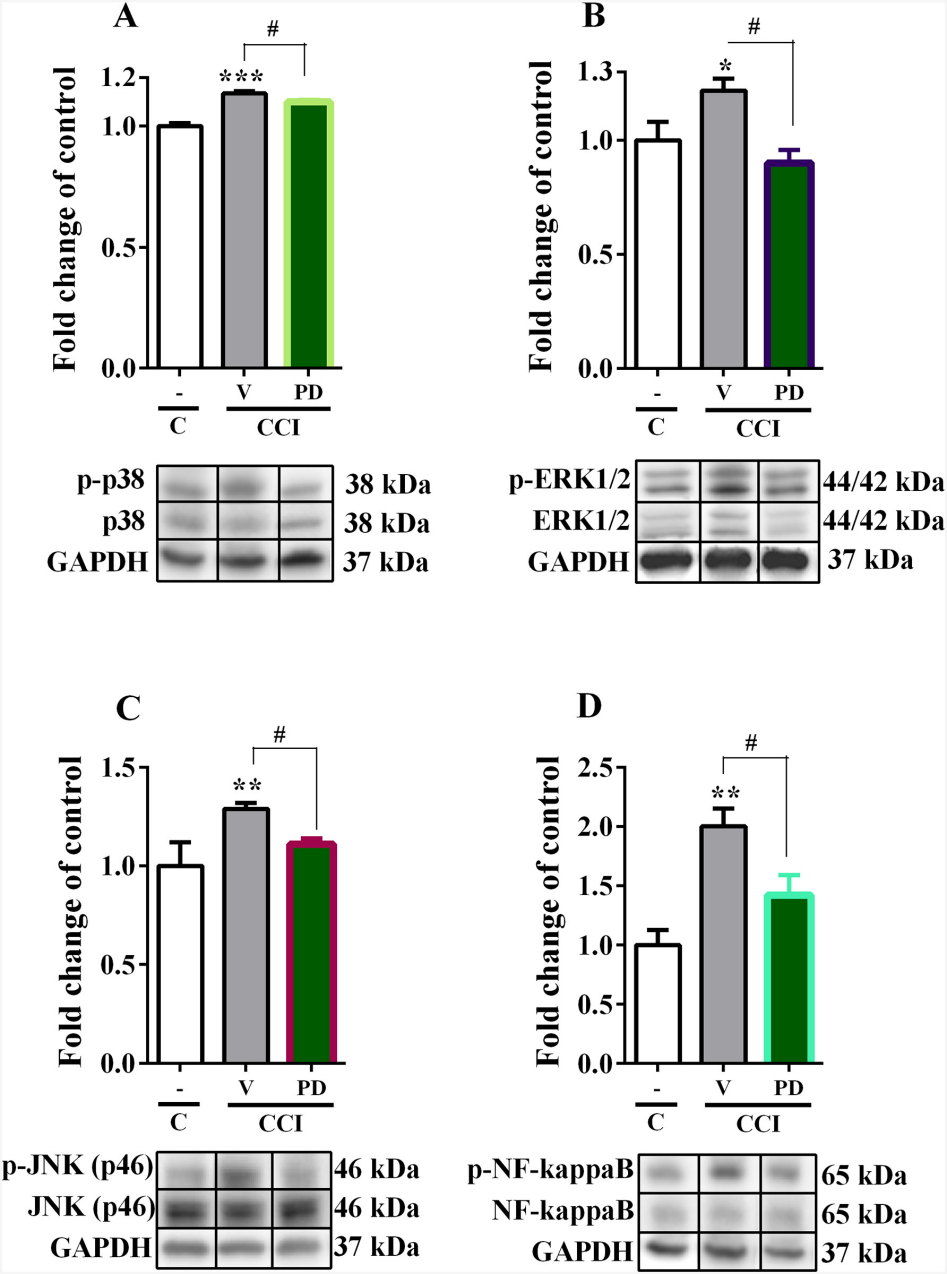
Effect of PD98059 on the p38, ERK1/2, JNK and NF-kappaB protein level in neuropathic pain. Effect of PD98059 (2.5 mcg/5 mcl; *i*.*t*.; 16 h and 1 h before CCI and then once daily for 7 days) on the protein levels of p38 (A), ERK1/2 (B), JNK (C) and NF-kappaB (D) in the ipsilateral dorsal part of the lumbar spinal cord on day 7 after CCI. PD98059 prevent the upregulation of the protein levels of p38, ERK1/2, JNK and NF-kappaB during neuropathic pain. The data are presented as the fold change of control. The number of individual samples per group Fig A-D: C n = 4–6; V-CCI n = 5–6; PD-CCI n = 5–7. The results for phosphorylated form of p38, ERK1/2, JNK and NF-kappaB were normalized to their total protein level in the same sample and were then expressed as a ratio of the average optical density values obtained for naïve animals. Inter-group differences were analyzed using Bonferroni's multiple comparison test. *p<0.05, **p<0.01 and ***p<0.001 indicates a significant difference compared to the control group (C), and ^#^p<0.05 indicate significant differences compared to the V-CCI rats (ANOVA, Bonferroni’s test). C, control; V, vehicle; PD, PD98059.

The protein levels of phosphorylated and non-phosphorylated p38, ERK1/2, JNK and NF-kappaB in the ipsilateral dorsal spinal cord (L4–L6) were examined seven days after CCI using Western blot technique. The p38 protein level (38kDa) was increased in vehicle-treated CCI-exposed rats and repeated PD98059 administration prevented its upregulation in neuropathic rats (Fig 2A). The increased level of ERK1/2 (44/42kDa) in CCI-exposed rats was not observed in group which received repeated administration PD98059 (Fig 2B). In case of JNK, the 46kDa isoform was upregulated in CCI-exposed rats (Fig 2C), however the changes of 54kDa JNK isoform was not detected (data not shown). Repeated administration of PD98059 resulted inhibition of upregulation of JNK 46kDa isoform in neuropathic rats (Fig 2C), and had no influence on JNK 54kDa isoform (data not shown). The NF-kappaB (65kDa) protein level was increased in CCI-exposed rats compared to control animals (Fig 2D) and repeated PD98059 administration prevented its upregulation in neuropathic animals (Fig 2D).

### The effect of repeated *i*.*t*. administration of PD98059 on spinal pronociceptive factors (IL-1beta, IL-6, IL-18 and iNOS) and antinociceptive factor (IL-10) on day seven after injury in CCI-exposed rats

**Fig 3:**
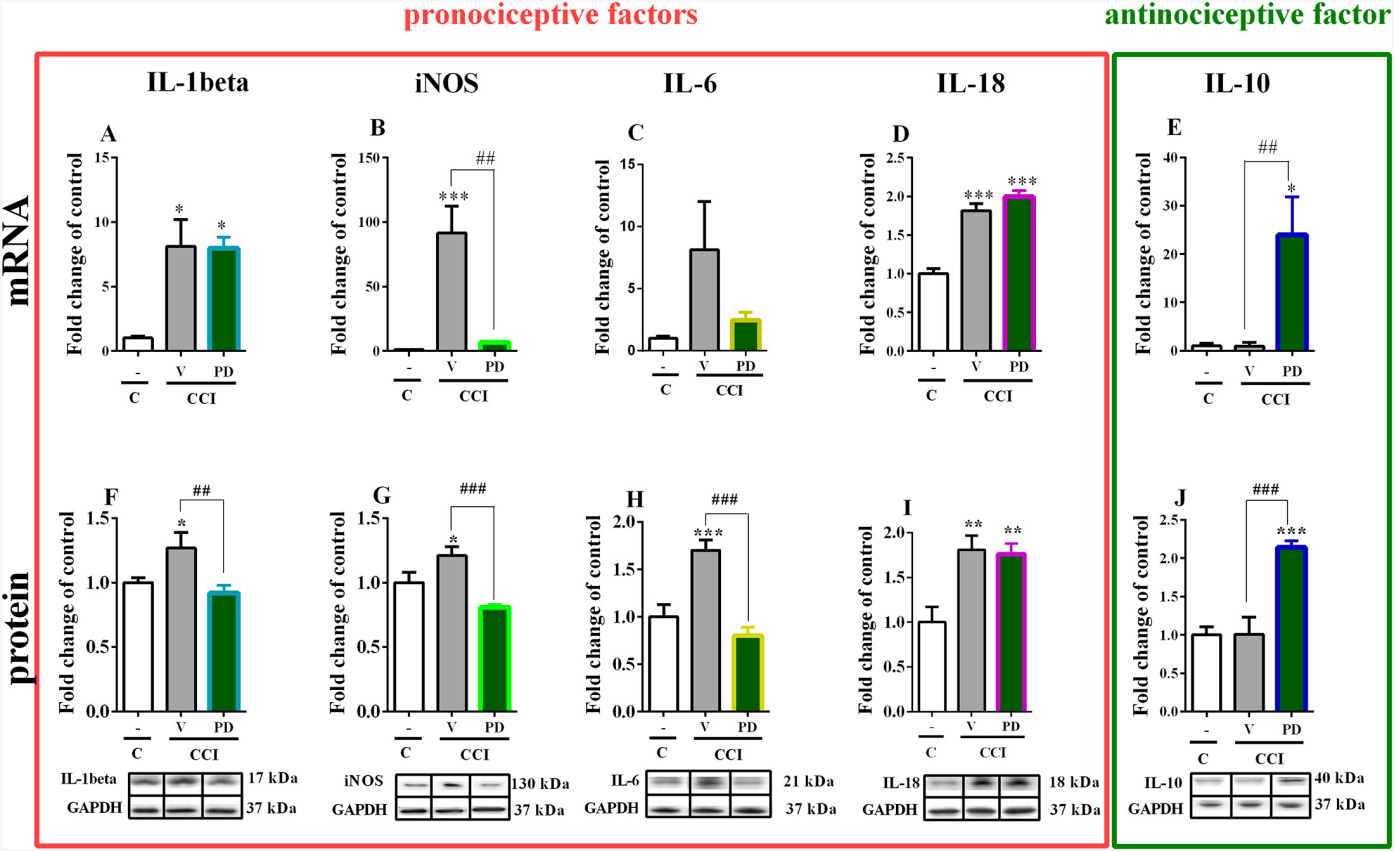
Effect of PD98059 on the mRNA and protein level of pro-inflammatory factors (IL-1beta, iNOS, IL-6 and IL-18) and anti-inflammatory factor (IL-10) in neuropathic pain. Effect of PD98059 (2.5 mcg/5 mcl; *i*.*t*.; 16 h and 1 h before CCI and then once daily for 7 days) on the mRNA and protein levels of IL-1beta (A, F), iNOS (B, G), IL-6 (C, H), IL-18 (D, I) and IL-10 (E, J) in the ipsilateral dorsal part of the lumbar spinal cord on day 7 after CCI. PD98059 prevent the upregulation of the mRNA levels of *iNOS* (B) and induced expression of mRNA for *IL-10* (E), but did not influence the level of IL-1beta (A), IL-6 (C) and IL-18 (D) in the spinal cord during neuropathic pain. Repeated PD98059 treatment prevent the upregulation of the protein levels of IL-1beta (F), iNOS (G), IL-6 (H) and induced IL-10 (J), but did not influence the level of IL-18 (I) in the spinal cord during neuropathic pain. The data are presented as the fold change of control. The number of individual samples per group Figs A-E: C n = 4–5; V-CCI n = 6; PD-CCI n = 4–5; Figs F-J: C n = 4–6; V-CCI n = 5–8; PD-CCI n = 6–7. Inter-group differences were analyzed using Bonferroni's multiple comparison test. *p<0.05, **p<0.01 and ***p<0.001 indicate significant differences compared to the control group (C), and ^##^p<0.01 and ^###^p<0.001 indicate significant differences compared to the V-CCI rats (ANOVA, Bonferroni’s test). C, control; V, vehicle; PD, PD98059.

The mRNA levels of *IL-1beta*, *IL-6*, *IL-18*, *iNOS* and *IL-10* in the ipsilateral dorsal spinal cord (L4–L6) were examined seven days after CCI using qRT-PCR assay. The level of *IL-1beta*, *iNOS* and *IL-18* were upregulated in CCI-exposed rats compared to control animals (Fig 3A, 3B and 3D) but the repeated PD98059 administration prevent increased of *iNOS* (Fig 3B) level. We observed in vehicle-treated CCI-exposed rats that the IL-6 (21 kDa) protein level was highly increased but the *IL-6* mRNA level shown only tendency to increase and the repeated administration of PD98059 had not significant effect on *IL-6* mRNA (Fig 3C).

Seven days following CCI the changes between mRNA levels of *IL-10* in control and CCI-exposed rats were not observed (Fig 3E). However, the repeated administration of PD98059 for seven days after CCI induced strong upregulation of *IL-10* mRNA level (Fig 3E).

The protein levels of IL-1beta, IL-6, IL-18, iNOS and IL-10 in the ipsilateral dorsal spinal cord (L4–L6) were examined seven days after CCI using Western blot technique. The level of IL-1beta (17 kDa) and iNOS (130 kDa) were similarly upregulated in CCI-exposed rats compared to control animals (Fig 3F and 3G) and the repeated PD98059 administration prevent increased of both IL-1beta (Fig 3F) and iNOS (Fig 3G) protein levels. We have showed that during neuropathic pain, PD98059 administration prevent upregulation of the spinal protein levels of IL-1beta, iNOS, IL-6, but not IL-18. Using qRT-PCR methods, we indicated that PD98059 administration prevent the upregulation of the spinal level of mRNA for *iNOS*, but not for *IL-1beta*, *IL-6* and *IL-18*. In vehicle-treated CCI-exposed rats the upregulation of IL-18 (18 kDa) protein level was observed and the repeated treatment with PD98059 had not influenced on the level of this interleukin (Fig 3I).

Seven days following CCI the changes between levels of IL-10 (40 kDa) in control and CCI-exposed rats were not observed (Fig 3J). However, the repeated administration of PD98059 for seven days after CCI induced strong upregulation of IL-10 protein level (Fig 3J).

### The effect of repeated *i*.*t*. administration of PD98059 on morphine analgesia on day seven after injury in CCI-exposed rats

**Fig 4:**
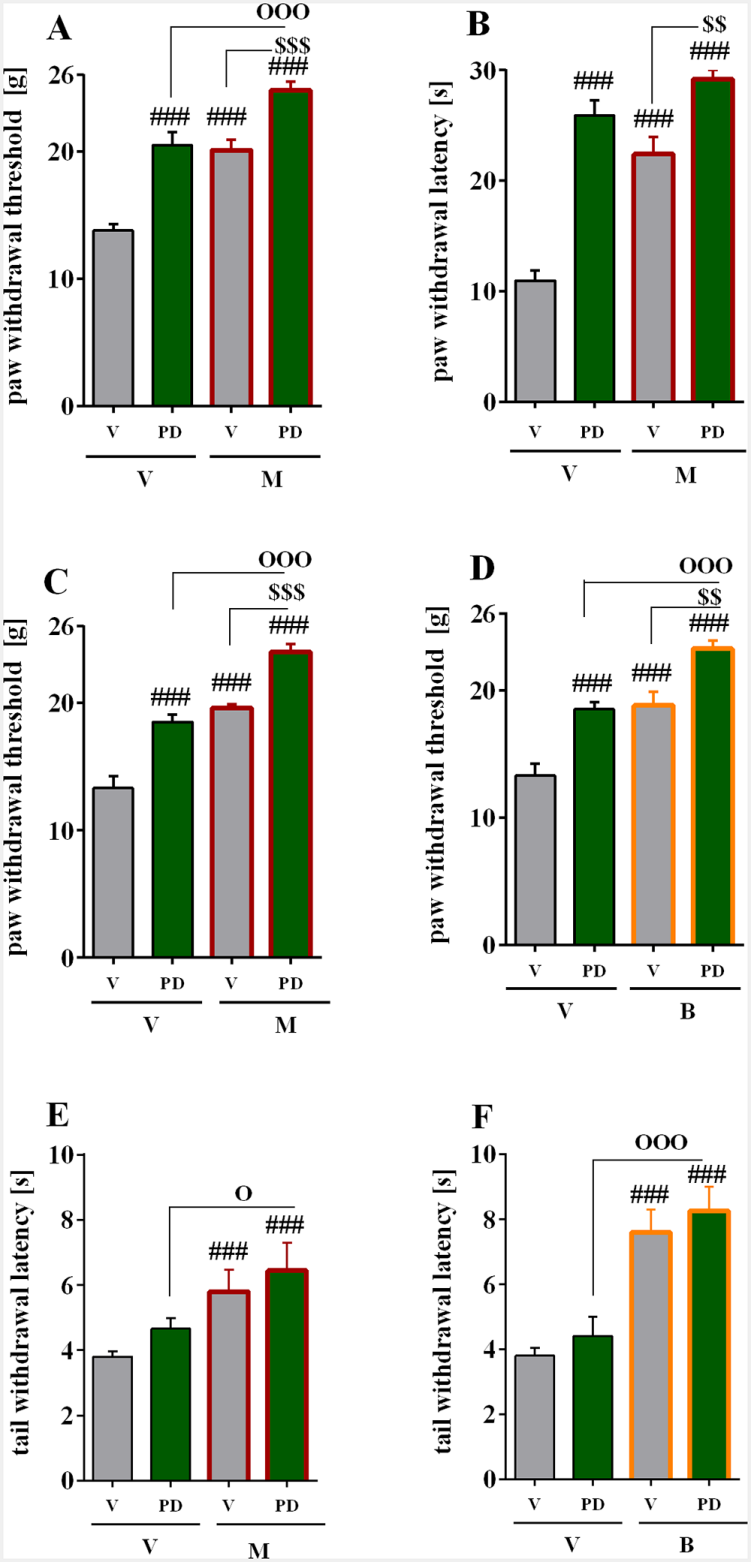
Effect of PD98059 on opioid analgesia in a naive and neuropathic rats. Effect of repeated vehicle or PD98059 administration (2.5 mcg/5 mcl; *i*.*t*.; 16 h and 1 h before CCI and then once daily for 7 days) on the analgesic effects of a single injection of morphine (5 mcg/5 mcl; *i*.*t*.) in CCI-exposed rats on day 7 after injury as measured by von Frey (A) and cold plate (B) in neuropathic animals. Effect of a single administration of vehicle or PD98059 (2.5 mcg/5 mcl) on a single *i*.*t*. injection of morphine (C, 2.5 mcg/5 mcl) and buprenorphine (D, 2.5 mcg/5 mcl) as measured by von Frey in neuropathic animals. Effect of a single administration of vehicle or PD98059 (2.5 mcg/5 mcl) on a single *i*.*t*. injection of morphine (E, 0.5 mcg/5 mcl) and buprenorphine (F, 2.5 mcg/5 mcl) as measured by tail flick test in naive animals. Behavioral tests were conducted 30 min after vehicle or PD98059 administration and then 30 min after a single vehicle, morphine or buprenorphine injection. Data are presented as the means ± SEM. The inter-group differences were analyzed using ANOVA and Bonferroni’s multiple comparison test. ^###^p<0.001 indicates a significant difference compared to the control group; ^$ $^p<0.01 and ^$ $ $^p<0.001 indicate significant differences compared to the repeated or single PD-treated CCI-exposed rats which received single morphine or buprenorphine injection, and °p<0.05, and °°°p<0.001 indicate significant differences between repeated or single PD-treated CCI-exposed or naive rats to repeated or single PD-treated CCI-exposed or naive rats which received single morphine or buprenorphine C, control; V, vehicle; PD, PD98059. The number of animals per group—Figs A&B: V+V-CCI n = 10; PD+V-CCI n = 10, V+M-CCI n = 10, PD+M-CCI n = 7; Fig C: V+V-CCI n = 10; PD+V-CCI n = 10, V+M-CCI n = 8, PD+M-CCI n = 8; Fig D: V+V-CCI n = 10; PD+V-CCI n = 10, V+M-CCI n = 8, PD+M-CCI n = 7; Fig E: V+V-CCI n = 12; PD+V-CCI n = 10, V+M-CCI n = 7, PD+M-CCI n = 6; Fig F: V+V-CCI n = 8; PD+V-CCI n = 12, V+B-CCI n = 6, PD+B-CCI n = 6.

In CCI-exposed rats repeated intrathecal PD98059 (2.5 mcg) administration produced after 30 min a strong anti-allodynic effect (20.5 g ± 1.0, n = 10) as measured on day seven compared to vehicle-treated animals (13.08 g ± 0.5, n = 10; Fig 4A). Single injection of morphine attenuated on allodynia (20.1 g ± 0.8, n = 10, Fig 4A). Repeated intrathecal PD98059 (2.5 mcg) administration improved the response to morphine (24.8 ± 0.7, n = 7, Fig 4A).

Similarly, repeated intrathecal PD98059 (2.5 mcg) administration produced potent anti-hyperalgesic effect (25.9 s ± 1.4, n = 10) on day seven after CCI compared to (10.96 s ± 0.9, n = 10) vehicle-treated CCI rats (Fig 4B). Single injection of morphine attenuated on hyperalgesia (22.43 g ± 1.5, n = 10, Fig 4B) and repeated intrathecal PD98059 (2.5 mcg) administration enhanced morphine-induced analgesia (29.2 s± 0.8, n = 7, Fig 4B).

### The effect of single *i*.*t*. administration of PD98059 on morphine and buprenorphine analgesia on day seven after injury in CCI-exposed rats

In CCI-exposed rats single intrathecal PD98059 (2.5 mcg) administration produced also significant antiallodynic effect after 30 min as assessed at day 7 by the von Frey test (18.5 g ± 0.55, n = 10; Fig 4C and 4D). Single injection of morphine attenuated the allodynia (19.6g ± 0.6, n = 8, Fig 4C). Single intrathecal PD98059 (2.5 mcg) administration enhanced morphine-induced analgesia (24.2 ± 0.63, n = 8, Fig 4C). Also single injection of buprenorphine produced analgesic effect as assessed by the cold plate test (18.8 ± 1.05, n = 8, Fig 4D). Single intrathecal PD98059 (2.5 mcg) administration enhanced buprenorphine-induced analgesia (23.25 ± 0.62, n = 7, Fig 4D).

### The effect of single *i*.*t*. administration of PD98059 on morphine and buprenorphine analgesia in naive rats

In naïve rats a single intrathecal injection of PD98059 (2.5 mcg) produced no significant analgesic effect as assessed by the tail flick test (V-treated 3.8 s ± 0.17, n = 12 vs. PD98059-treated 4.6 s ± 0.32, n = 10; Fig 4E and 4F). Single intrathecal morphine (0.5 mcg) administration produced antinociceptive effect (5.8 s ± 0.66, n = 7, Fig 4E). Also single injection on buprenorphine (2.5 mcg) produced analgesic effect (7.6 s ± 0.7, n = 6, Fig 4F). However, a single intrathecal administration of PD98059 did not potentiated morphine (Fig 4E) and buprenorphine-induced (Fig 4F) analgesia as measured in tail-flick test 30 min after opioids *i*.*t*. administration.

## Discussion

In the present paper we have demonstrated that PD98059 administration can diminish chronic constriction sciatic nerve injury-induced allodynia and hyperalgesia 3 and 7 days after CCI. Similar to our study, Zhuang et al., in 2005 [8] presented that a single injection of PD98059 into the lumbar CSF space inhibited spinal nerve ligation-induced (SNL)-induced mechanical allodynia. In 2014, van den Heuvel [33] et al. demonstrated that the intrathecal administration of PD98059 significantly reduced mechanical hyperalgesia in rats after plantar surgical incision. In mice, intraperitoneal administration of PD98059 (10 mg/kg) reduced inflammation in a carrageenan pain model [34]. Alessandrini et al. [35] observed that treatment of mice with PD98059 before focal cerebral ischemia provides protection against damage. Clemons et al. have shown that PD98059 can also significantly relieve cerulein-induced acute pancreatitis [36]. Our results show for the first time that PD98059 has not only analgesic effects, but also potentiates morphine and buprenorphine analgesia. Our Western blot studies have shown that PD98059, an inhibitor of MEK1/2, downregulated the CCI-elevated p-ERK1/2, as well as p-p38, p-JNK and p-NF-kappaB protein levels in the spinal cord in neuropathy. Moreover, PD98059 shifts the balance from a pro-inflammatory (IL-1beta, IL-6, and iNOS) to anti-inflammatory factors (IL-10).

Release of both pro- and anti-nociceptive cytokines by activated spinal immune and immune-like glial cells induce neuropathic pain which therefore is now considered as a neuro-immune disorder. Many of the changes occurring after tissue injury attempt to restore homeostasis in the damaged tissue, but lead to an altered balance that underlies ongoing chronic pain. Molecular components of the intracellular signaling cascades responsible for sensitization have been proposed as key targets for pharmacological treatments. Many signaling pathways can interact with each other through intracellular cross-talk. The results of our study suggest that MEKs (kinase enzymes that phosphorylate MAPKs) are useful targets because PD98059, an inhibitor of MEK1/2, can influence a variety of cell signaling pathways that are involved in neuropathic pain states (S4 Fig). Some inhibitors of ERK, p38, and JNK have been previously shown to reduce neuropathic pain symptoms in different animal models [13, 20, 25, 37, 38, 39, 40, 41].

In a neuropathic pain model the successive activation of ERK in spinal neurons, then in microglia, and finally in astrocytes was described [8, 42 4, 43 4]. Activation of ERK in spinal cord dorsal horn neurons is associated with the activation of nociceptive-specific sensory fibers and the promotion of intracellular events that contribute to central sensitization, which may be manifested at both behavioral and cellular levels [44]. Studies conducted by Xu et al. in 2006 [45] demonstrated enhanced activation of ERK1/2 and p38 after spinal cord injury in rats, which is consistent with our results. Furthermore, we indicate that repeated PD98059 administration prevents spinal upregulation of ERK1/2 and p38 during neuropathy, which suggests that PD98059 induce substantial changes in the intracellular pathways in cells. Zhuang et al. in 2005 [8] indicated that in SNL model the anti-allodynic effect of intrathecal PD98059 may be partially mediated by reducing ERK activation in DRG cells. All this studies suggested that ERK inhibitors may have a useful role in the management of neuropathy.

Some studies indicate that the active form of spinal JNK—pJNK increases after nerve injury [20, 37]. In a study by Zhuang et al. [20] the authors showed that only the p-JNK 46kDa isoform, but not p-JNK 54kDa isoform, is present in the spinal cord after injury, which corresponds well with our results. It has been previously shown that intrathecal infusion of JNK inhibitor, SP600125, attenuates neuropathic pain [1, 20]. We have shown for the first time that intrathecal delivery of PD98059 prevents upregulation of JNK 46kDa isoform level, so our results suggest that its influence on JNK can be one of the reason of pain relief.

Interestingly, in contrast to selective p38, ERK or JNK inhibitors, which often failed to reverse established pain states [1, 20, 41], PD98059 seems to be more effective. This specific MEK inhibitor appears inhibit a wide range of actions, and, most importantly, we found that it was also effective after a single injection when neuropathic pain was well established on day 7 after the injury.

It is well known that the p38 pathway phosphorylates and enhances the activity of many transcription factors, including NF-kappaB and the NF-kappaB regulates the transcription of various genes, including cytokines and iNOS [27, 42]. A number of studies indicate that NF-kappaB is involved in the pathogenesis of neuropathic pain. NF-kappaB activation occurs in the spinal cord after peripheral nerve injury, which is in agreement with our results and the findings of other authors [22, 23, 24]. In our previously published paper [25], chronic treatment with parthenolide (an inhibitor of NF-kappaB) showed that inhibition of the NF-kappaB pathway produced strong analgesic effects. Downregulation of NF-kappaB activation after parthenolide treatment was correlated with reduction of pronociceptive factor (IL-1beta, IL-18 and iNOS) at the spinal cord level of CCI-exposed rats [46]. The effect obtained by PD98059 treatment was similar in the case of allodynia, but stronger in the case of thermal hyperalgesia. Interestingly, using Western blot analysis, we showed that PD98059 administration prevents upregulation of NF-kappaB increased after CCI. Those findings suggests that NF-kappaB activation is one of the mechanisms of analgesia during neuropathic pain.

It is now known that the regulation of cytokine biosynthesis in many cell types is mediated through the activation of MAPK family members (JNK, p38, and ERK1/2). PD98059 diminished the three MAPK signal transduction pathways and the activity of NF-kappaB, and as consequence, modulated nerve injury-induced cytokine changes. Interleukins are potent modulators of pain cascades on the spinal cord level after nerve injury, inducing pain through direct neuronal excitability. Other effects of cytokines include changes in intracellular MAPK signaling cascades, resulting in recruitment of macrophage and activation of glia. It is well documented that p38 plays a role in neuropathic pain, and this effect is correlated with microglia activation [13, 14, 16]. Strategies that lead to a reduction in pain include the inhibition of pronociceptive factors. In our recent paper [46] we have demonstrated that upregulated after CCI pronociceptive factors (like IL-1beta, IL-18, iNOS) at the spinal cord level are microglial origin and are correlated with p38MAPK and ERK1/2, *inter alia*, activation. Zhou et al. [47] have shown that downregulation of IL-1beta, IL-6 and TNFalpha was correlated with reduction of activated p38MAPK level after montelukast-treatment in CCI-exposed rats. It has been shown, that PD98059 intracisternally administered after the subcutaneous injection of interleukin-1beta attenuated the formalin-induced pain behavior [46]. These results suggest that the IL-1beta-induced central sensitization of nociception is mediated by the spinal MAPK pathways, which are activated in neurons and glia under pain conditions. The nociceptive factors that undergo the strongest activation during neuropathy include iNOS, IL-1beta, IL-18 and IL-6 [16, 31, 48–54]. We showed that during neuropathic pain, PD98059 administration prevented the upregulation of the spinal protein levels of IL-1beta, iNOS, IL-6, but not IL-18 what constitutes the novelty of this research. Using qRT-PCR methods, we indicated that PD98059 administration prevented the upregulation of the spinal level of mRNA for *iNOS*, but not for *IL-1beta*, *IL-6* and *IL-18*. Similar to our qRT-PCR findings, Xu et al. [47] show that PD98059 attenuated the spinal injury-induced expression of *iNOS* mRNA. However, no studies currently show how PD98059 influences interleukin changes under pathological states *in vivo*. In 2014, Zhu et al. [55] demonstrated that PD98059 slightly decreased IL-6 in LPS-stimulated murine BV2 microglial cells *in vitro*. Several studies have shown that PD98059 suppresses IL-1beta-induced phosphorylation of Erk1/2 in rat C6 gliomas; however, it does not decrease IL-6 [56, 57]. In 2013, Meng et al. [58] demonstrated that PD98059 diminishes the LPS-induced overexpression of NO in rat vascular smooth muscle cells *in vitro*. In 1998, Foey et al. [59] proved that PD98059 inhibited LPS-induced IL-1beta in monocyte cell culture, nevertheless, it performed no influence on LPS-induced IL-10 production. On the contrary to results mentioned, PD98059 inhibited IL-10 secretion by LPS-activated macrophages [60].

Our behavioral studies show that PD98059 prevented the upregulation of spinal protein level of IL-1beta that occurs following nerve injury and in contrast, induced the upregulation of IL-10. Studies conducted by Milligan et al. [61] and Ledeboer et al. [62] have shown that intrathecal administration of IL-10 suppresses neuropathic pain symptoms. The role of IL-10 in the nervous system is still not explained and many authors hypothesized that it may interact with microglia by preventing the release of IL-6 and IL-1beta [52, 63, 64]. IL-10 is an antinociceptive cytokine and PD98059 significantly increased the level of this cytokine. Therefore, in our opinion PD98059 restores the neuroimmune balance that is biased towards pronociceptive factors in the development of neuropathic pain.

The most important, new aspect of this study is that repeated and single administration of PD98059 potentiates the analgesic effect of single morphine and/or buprenorphine in CCI-exposed rats. Increasing evidence indicates that chronic morphine induces cytokine activation [12, 18, 65, 66] and that MAPKs are involved in agonist-induced phosphorylation of the opioid receptors [67]. In 2010, Wang et al. [68] showed that p-p38 was upregulated following chronic morphine treatment, other authors have shown that inhibition of p38 attenuated the development of morphine tolerance [12, 68]. We also demonstrated that minocycline antagonizes morphine antinociceptive tolerance during neuropathic pain [18]. In 2014, we hypothesized [25] that the modulation of NF-kappaB signaling during neuropathic pain may be one of the key factors associated with the loss of opioid effectiveness. *In vitro* studies have confirmed that NF-kappaB is involved in the transcriptional regulation of *mor* [69], *dor* [70] and *kor* [71] gene expression. It was shown previously that the ERK signaling cascade may be initiate by the Ca^2+^ entry into neurons via ionotropic glutamate receptors [72, 73]. In the paper of Ji et al. [7] the authors analyzed the involvement of ERK in producing pain hypersensitivity in formalin-induced pain model. In the mention work authors investigated that NMDA receptors contribute to ERK activation in the dorsal horn while MK-801, a blocker of the NMDA receptor channel, partially evoked ERK activation. Kawasaki et al. [39] suggested that the phosphorylation of the signaling molecule ERK could be a useful tool for studying neuronal sensitivity to opioid treatment under nerve injury conditions. We demonstrated in our earlier study [25] that high selective MEK1/2 inhibitor (U0126) significantly upregulated mRNA for classical opioid receptors, *MOR*, *KOR* and *DOR* at the spinal cord level 7 days after CCI. Those changes in opioid receptors expression may contribute to better analgesic effects of morphine and buprenorphine. Chronic morphine treatments induce the spinal activation of MEK1/2 and may be responsible for the altered properties of morphine [74]. In our experiments [25], we have shown that the chronic, intrathecal administration of selective inhibitor of MEK1/2 (U0126) improves the effectiveness of morphine in neuropathic pain, however those effect was weaker than in case of PD98059. The binding sites for PD98059 and U0126 on MEK1 appear to overlap but their mechanisms of action seem to be different [75, 76]. The differences between those two MEKs inhibitors also are manifested in specificity and potency [76, 77], which may explain better effects of PD98059.

Our results indicate that inhibition of the MAPK pathways by PD98059 has significant analgesic effects. The PD98059 robustly prevents the p38, ERK, JNK and NF-kappaB activation induced by nerve injury, and therefore, contributes to increasing the effectiveness of opioids such as morphine and buprenorphine in neuropathy. The data presented suggest that the blockade of the MAPKK pathways can be a potential target for a new and more successful therapeutic treatment for neuropathic pain.

## Supporting Information

S1 Fig Drug administration. (DOCX)

S2 Fig Amplification plot of *HPTR* transcripts. (DOCX)

S3 Fig The full length of an exemplary immunoblot. (DOCX)

S4 Fig The PD98059, an inhibitor of MEK1/2, induced changes during neuropathic pain. (DOCX)

S1 Table Drug used in the study. (DOCX)

S2 Table Primers used in the study. (DOCX)

S3 Table Antibodies used in the study. (DOCX)
