# Supplementary material for: PD98059 Influences Immune Factors and Enhances Opioid Analgesia in Model of Neuropathy
Source: PLoS One. 2015 Oct 1;10(10):e0138583. doi: 10.1371/journal.pone.0138583 (PMC4591269; doi:10.1371/journal.pone.0138583)
Supplement: S1 Fig — (DOCX) [file pone.0138583.s001.docx]

S1 Fig. Drug administration
