# Supplementary material for: PD98059 Influences Immune Factors and Enhances Opioid Analgesia in Model of Neuropathy
Source: PLoS One. 2015 Oct 1;10(10):e0138583. doi: 10.1371/journal.pone.0138583 (PMC4591269; doi:10.1371/journal.pone.0138583)

**S2 Fig. Amplification plot of *HPTR* transcripts.** *HPRT* transcript levels do not significantly change in CCI-exposed rats, therefore, served as an adequate housekeeping gene. In V-treated (0.99 ± 0.005, n=4) and PD98059-treated (0.99 ± 0.004, n=6) CCI-exposed rats the mRNA levels of HPRT in the ipsilateral dorsal spinal cord (L4-L6) were unchanged as compare to the control (naïve) (1 ± 0.003, n=6). The data are presented as fold change of control. HPRT transcript levels do not significantly change in CCI-exposed rats, therefore, served as an adequate housekeeping gene [16, 17, 18, 31]. We inserted an amplification plot for HPRT transcripts from our present experiments.


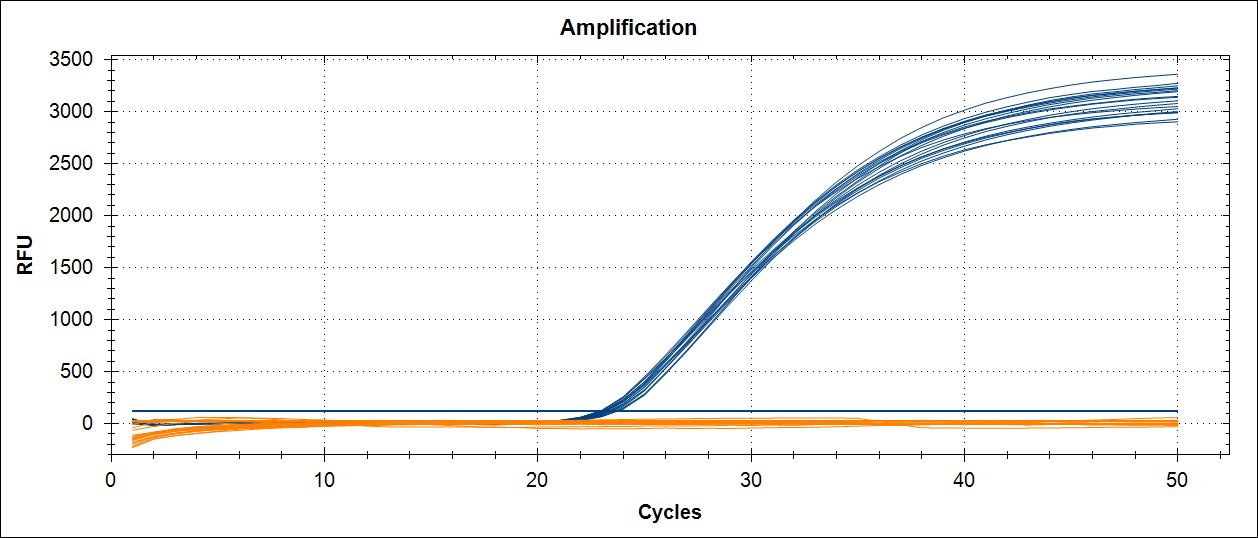

Supplement: S2 Fig — (DOCX) [file pone.0138583.s002.docx]
