# Supplementary material for: PD98059 Influences Immune Factors and Enhances Opioid Analgesia in Model of Neuropathy
Source: PLoS One. 2015 Oct 1;10(10):e0138583. doi: 10.1371/journal.pone.0138583 (PMC4591269; doi:10.1371/journal.pone.0138583)
Supplement: S3 Fig — (DOCX) [file pone.0138583.s003.docx]

S3 Fig. The full length of an exemplary immunoblot:


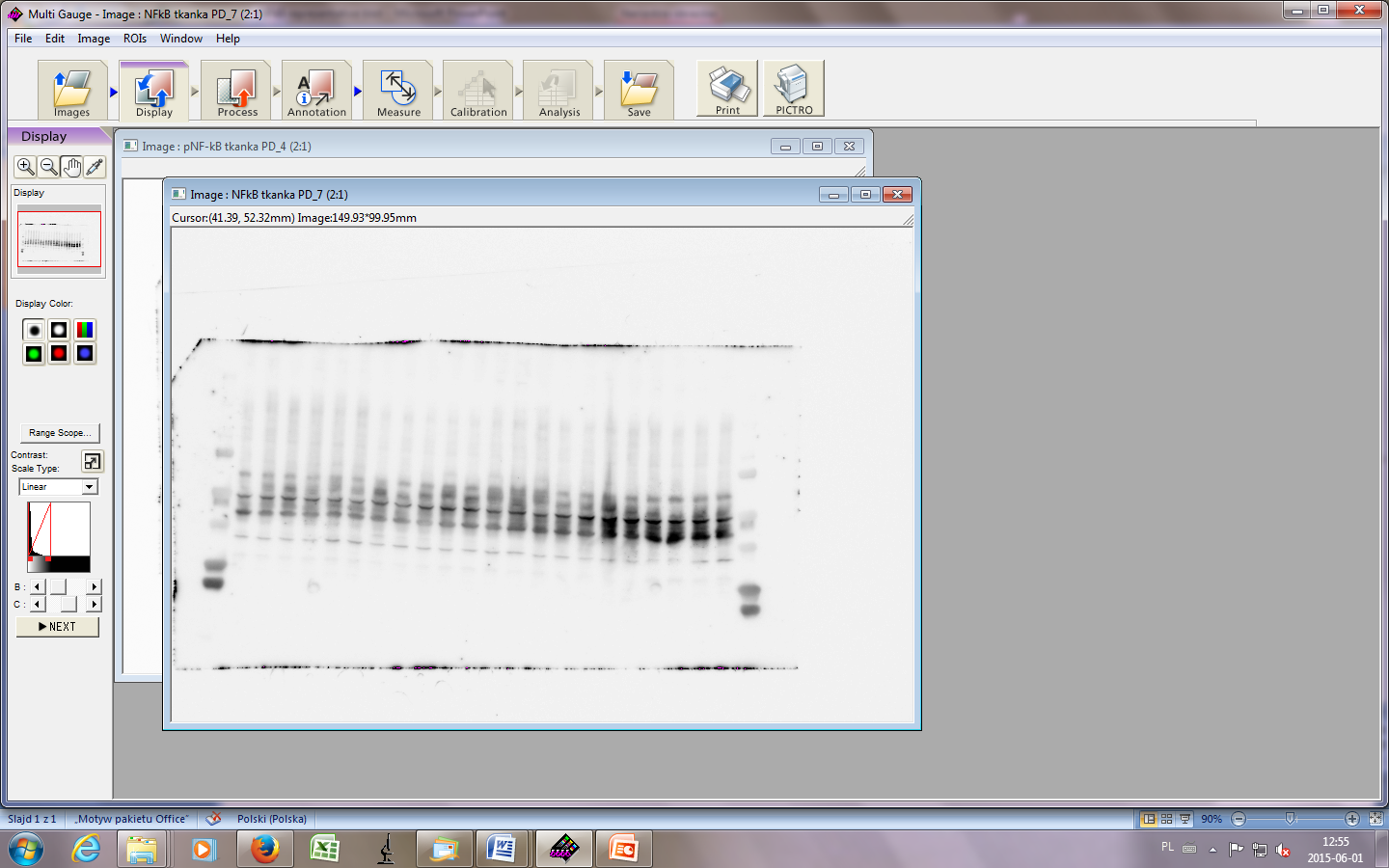

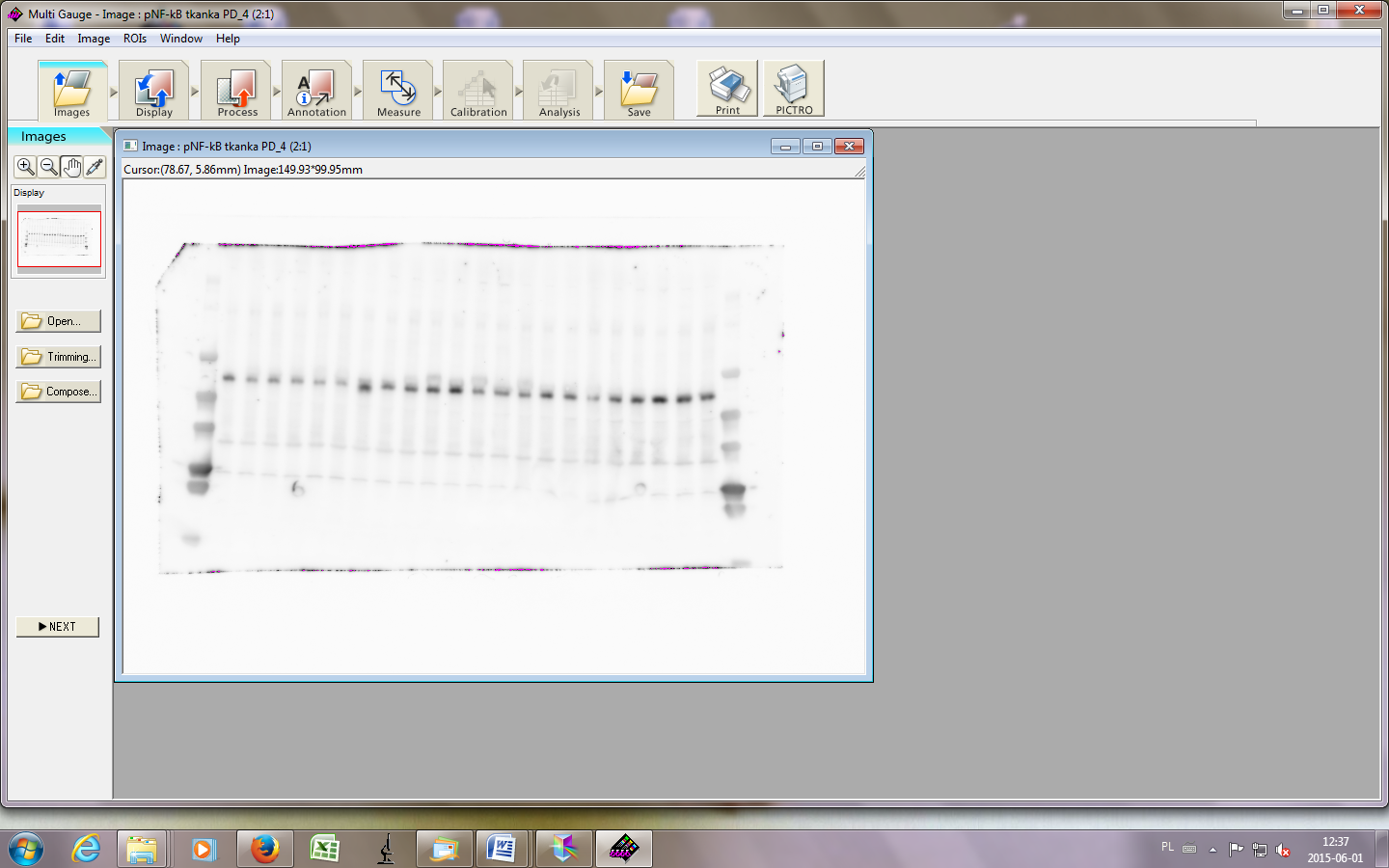


intact

vehicle-CCI

PD-CCI

**p-NF-κB**

**NF-κB**

**GAPDH**

**65 kDa**

**65 kDa**

**37 kDa**
