# Supplementary material for: PD98059 Influences Immune Factors and Enhances Opioid Analgesia in Model of Neuropathy
Source: PLoS One. 2015 Oct 1;10(10):e0138583. doi: 10.1371/journal.pone.0138583 (PMC4591269; doi:10.1371/journal.pone.0138583)
Supplement: S4 Fig — (DOCX) [file pone.0138583.s004.docx]

S4 Fig. The PD98059, an inhibitor of MEK1/2, induced changes during neuropathic pain. PD98059: 1) downregulated the CCI-elevated ERK1/2, as well as p38, JNK and NF-kappaB protein levels in neuropathy; 2) restored equilibrium between algesic (IL-1beta, IL-6, and iNOS) and analgesic factors (IL-10); 3) diminished pain; 4) increased the effectiveness of morphine and buprenorphine in neuropathy.
