# Supplementary material for: PD98059 Influences Immune Factors and Enhances Opioid Analgesia in Model of Neuropathy
Source: PLoS One. 2015 Oct 1;10(10):e0138583. doi: 10.1371/journal.pone.0138583 (PMC4591269; doi:10.1371/journal.pone.0138583)
Supplement: S1 Table — (DOCX) [file pone.0138583.s005.docx]

**S1 Table. Drug used in the study.**

| DRUG | DOSE | COMPANY |
| --- | --- | --- |
| PD98059 | 2.5 mcg/5 mcl | Sigma-Aldrich, USA |
| morphine | 0.5 or 2.5 mcg/5 mcl | Polfa Kutno, Poland |
| buprenorphine | 2.5 mcg/5 mcl | Polfa Waszawa, Poland |
