# Supplementary material for: PD98059 Influences Immune Factors and Enhances Opioid Analgesia in Model of Neuropathy
Source: PLoS One. 2015 Oct 1;10(10):e0138583. doi: 10.1371/journal.pone.0138583 (PMC4591269; doi:10.1371/journal.pone.0138583)
Supplement: S2 Table — (DOCX) [file pone.0138583.s006.docx]

**S2 Table. Primers used in the study.**

| **gene** | **name** | **number** | **company** | **catalog number** |
| --- | --- | --- | --- | --- |
| ***HPRT1*** | hypoxanthine guanine phosphoribosyl transferase | Rn01527838 g1 | Applied Biosystems | 4331182 |
| ***IL-1beta*** | interleukin 1 beta | Rn00580432_m1 |  |  |
| ***IL-6*** | interleukin 6 | Rn00561420_m1 |  |  |
| ***IL-18*** | interleukin 18 | Rn01422083_m1 |  |  |
| ***iNOS*** | inducible nitric oxide synthase | Rn00561646_m1 |  |  |
| ***IL-10*** | interleukin 10 | Rn00563409_m1 |  |  |
