# Supplementary material for: PD98059 Influences Immune Factors and Enhances Opioid Analgesia in Model of Neuropathy
Source: PLoS One. 2015 Oct 1;10(10):e0138583. doi: 10.1371/journal.pone.0138583 (PMC4591269; doi:10.1371/journal.pone.0138583)
Supplement: S3 Table — (DOCX) [file pone.0138583.s007.docx]

**S3 Table. Antibodies used in the study.**

| **ANTIBODIES** | **DILUTION** | **COMPANY** | **CATALOG NUMBER** | **LINKS** |
| --- | --- | --- | --- | --- |
| **p-p38** | 1:500 | Santa Cruz | sc-101759 | <http://www.scbt.com/datasheet-101759-p-p38-thr-180-tyr-182-antibody.html> |
| **p38** | 1:500 | Santa Cruz | sc-7149 | <http://www.scbt.com/datasheet-7149-p38alpha-beta-h-147-antibody.html> |
| **p-ERK1/2** | 1:500 | Santa Cruz | sc-13900 | <http://www.scbt.com/datasheet-13900-npas2-k-17-antibody.html> |
| **ERK1/2** | 1:500 | Santa Cruz | sc-16982 | <http://www.scbt.com/datasheet-16982-p-erk-1-2-thr-202-tyr-204-antibody.html> |
| **p-JNK** | 1:2000 | Santa Cruz | sc-12882 | <http://www.scbt.com/datasheet-12882-p-jnk-thr-183-tyr-185-antibody.html> |
| **JNK** | 1:2000 | Santa Cruz | sc-7345 | <http://www.scbt.com/datasheet-7345-jnk-d-2-antibody.html> |
| **p-NF-kappaB** | 1:500 | Santa Cruz | sc-33039 | <http://www.scbt.com/datasheet-33039-p-nfkappab-p65-ser-311-r-antibody.html> |
| **NF-kappa** | 1:500 | Santa Cruz | sc-372 | <http://www.scbt.com/datasheet-372-nfkappab-p65-c-20-antibody.html> |
| **IL-1beta** | 1:1000 | Abcam | Ab9787 | <http://www.abcam.com/il1-beta-antibody-ab9787.html> |
| **IL-6** | 1:1000 | Invitrogen | ARC0062 | <https://www.lifetechnologies.com/order/genome-database/antibody/IL-6-Antibody-Polyclonal/ARC0062> |
| **iNOS** | 1:2000 | Sigma-Aldrich | N7782 | <http://www.sigmaaldrich.com/catalog/product/sigma/n7782?lang=pl&region=PL> |
| **IL-18** | 1:1000 | R&D Systems | AF521 | <https://www.rndsystems.com/products/rat-il-18-il-1f4-antibody_af521> |
| **IL-10** | 1:2000 | Invitrogen | ARC0102 | <https://www.lifetechnologies.com/order/genome-database/antibody/IL-10-Antibody-Polyclonal/ARC0102> |
| **GAPDH** | 1:5000 | Millipore | MAB374 | <http://www.merckmillipore.com/PL/pl/product/,MM_NF-MAB374> |
